# Development of an Individualized Responsive Feeding Intervention—Learning Early Infant Feeding Cues: Protocol for a Nonrandomized Study

**DOI:** 10.2196/44329

**Published:** 2023-02-28

**Authors:** Jessica Bahorski, Mollie Romano, Julie May McDougal, Edie Kiratzis, Kinsey Pocchio, Insu Paek

**Affiliations:** 1 College of Nursing Florida State University Tallahassee, FL United States; 2 School of Communication Science and Disorders Florida State University Tallahassee, FL United States; 3 Center for Prevention & Early Intervention Policy Florida State University Tallahassee, FL United States; 4 College of Health and Human Sciences Florida State University Tallahassee, FL United States

**Keywords:** responsive feeding, infant growth, infant nutrition

## Abstract

**Background:**

Responsive infant feeding occurs when a parent recognizes the infant’s cues of hunger or satiety and responds promptly to these cues. It is known to promote healthy dietary patterns and infant weight gain and is recommended as part of the Dietary Guidelines for Americans. However, the use of responsive infant feeding can be challenging for many parents. Research is needed to assist caregivers recognize infant hunger or satiety cues and overcoming barriers to using responsive infant feeding.

**Objective:**

The Learning Early Infant Feeding Cues (LEIFc) intervention was designed to fill this gap by using a validated coaching approach, SS-OO-PP-RR (“super,” Setting the Stage, Observation and Opportunities, Problem Solving and Planning, Reflection and Review), to promote responsive infant feeding. Guided by the Obesity-Related Behavioral Intervention Trials model, this study aims to test the feasibility and fidelity of the LEIFc intervention in a group of mother-infant dyads.

**Methods:**

This pre-post quasi-experimental study with no control group will recruit mothers (N=30) in their third trimester (28 weeks and beyond) of pregnancy from community settings. Study visit 1 will occur prenatally in which written and video material on infant feeding and infant hunger and satiety cues is provided. Demographic information and plans for infant feeding are also collected prenatally via self-report surveys. The use of responsive infant feeding via subjective (survey) and objective (video) measures is recorded before (study visit 2, 1 month post partum) and after (study visit 5, 4 months post partum) intervention. Coaching on responsive infant feeding during a feeding session is provided by a trained interventionist using the SS-OO-PP-RR approach at study visits 3 (2 months post partum) and 4 (3 months post partum). Infant feeding practices are recorded via survey, and infant weight and length are measured at each postpartum study visit. Qualitative data on the LEIFc intervention are provided by the interventionist and mother. Infant feeding videos will be coded and tabulated for instances of infant cues and maternal responses. Subjective measures of responsive infant feeding will also be tabulated. The use of responsive infant feeding pre-post intervention will be analyzed using matched *t* tests. Qualitative data will be examined to guide intervention refinement.

**Results:**

This study initially began in spring 2020 but was halted because of the COVID-10 pandemic. With new funding, recruitment, enrollment, and data collection began in April 2022 and will continue until April 2023.

**Conclusions:**

After refinement, the LEIFc intervention will be tested in a pilot randomized controlled trial. The long-term goal is to implement LEIFc in the curricula of federally funded maternal-child home visiting programs that serve vulnerable populations—those that often have infant feeding practices that do not align with recommendations and are less likely to use responsive infant feeding.

**International Registered Report Identifier (IRRID):**

DERR1-10.2196/44329

## Introduction

Infant feeding practices, which include what and how infants are fed, contribute to infant growth and development, and thus, lifelong health [1]. Specifically, healthy infant feeding practices contribute to a healthy weight gain trajectory, which has been associated with decreased obesity later in life [2,3]. Healthy infant feeding practices include not only *what* infants are fed but also *how* infants are fed. The introduction of breastfeeding, breastfeeding until at least 6 months of age, introduction of complementary foods around 6 months and not before 4 months of age, and avoidance of juice and sugar sweetened beverages are recommended examples of *what* infants should be fed [1,4]. The responsive feeding approach is recommended for *how* infants should be fed and has been associated with healthy infant weight gain trajectories [4].

Responsive feeding occurs when a parent (or other caregiver) learns and recognizes the infant’s cues of hunger and satiety (Textbox 1) and responds promptly to these cues [5]. Responsive feeding is thought to promote the ability to recognize and respond to internal cues of hunger and satiety [6-10]. This ability to recognize these internal cues and self-regulate intake in response to physiologic need is associated with healthy weight gain during infancy as well as dietary behaviors later in life [8,11]. For these reasons, it is important that parents use the responsive feeding approach when feeding their infant.

**Hunger and satiety cues in infants less than 6 months [5,7].**

**Hunger signals**

- Wakes up and increased alertness
- Moves around
- Smacks lips
- Sucks on fist and fingers
- Cries or fusses
- Opens mouth wide
- Response to rooting reflex

**Satiety signals**

- Seals lips
- Turns head away
- Decreases or stops sucking
- Spits out nipple
- Falls asleep
- Decreased muscle tone

Three things must be in place for responsive feeding to occur: (1) the child signals hunger; (2) the caregiver recognizes cues and responds promptly; and (3) the child experiences a predictable response to hunger [5]. For this process to occur, caregivers must read their child’s nonverbal cues and respond contingently. The degree to which a caregiver responds contingently to a child’s communication, whether it is to a child’s hunger cues or to bids for social interaction, is widely known to predict a child’s language development [12-14]. As such, coaching caregivers to respond to feeding cues could impact overall caregiver responsiveness to their child’s communication bids, which could support the child’s early language development by extension.

Interventions to promote responsive feeding have shown benefit in preventing rapid infant weight gain and promoting healthy dietary patterns in infants and young children [15-22]. Despite the correlation between responsive feeding and healthy infant weight gain, the successful use of responsive feeding by caregivers is still lacking [23]. Caregivers report that recognition of hunger and satiety cues in their infant is challenging [24,25]. Infant cues can be subtle, and it can be difficult for parents to differentiate reflexive movements such as hand to mouth and rooting. Additionally, recent literature has identified maternal characteristics such as infant feeding beliefs, age, mental health, and the mother’s own eating behavior as contributing to the use of responsive feeding [26-29]. Infant temperament and support from health care professionals and family and friends also play a role [23,26]. Therefore, more research is needed to find ways to assist caregivers in recognition of hunger and satiety cues and to overcome additional barriers to promote the use of responsive feeding during infancy.

The Learning Early Infant Feeding Cues (LEIFc) intervention was designed to fill this gap by using a validated coaching approach with the mothers of new infants to promote responsive feeding. The proposed study aims to test the feasibility and fidelity of the LEIFc intervention in a group of mother-infant dyads. We aimed to (1) develop, refine, and test a responsive feeding intervention that can be used within real-world settings to serve mother-infant dyads in the community and (2) design the model with a primary emphasis on responsive feeding, but with a secondary focus on early communication to help caregivers understand the dual benefit of noticing and responding to infant cues.

## Methods

### Ethics Approval

The institutional review board at Florida State University approved this study (STUDY00002895).

### Theoretical Framework

The Obesity-Related Behavioral Intervention Trials (ORBIT) model is used to guide the proposed study [30]. The ORBIT model uses an iterative process and was chosen because the LEIFc intervention is behavioral in nature, in the early stage of development, and focused on prevention of a chronic disease [30]. Testing fidelity and feasibility of the LEIFc intervention study falls into phase I of the ORBIT model as the intervention has been designed but needs to be refined based on preliminary testing. The findings from this initial study will provide the foundation to move to phase II and conduct a proof-of-concept trail.

### Study Design and Intervention

This study uses a pre-post quasi-experimental design with no control group to examine the feasibility, fidelity, and social validity of the LEIFc intervention. The intervention was developed based on existing literature regarding enablers and barriers to the use of responsive feeding by caregivers and communication cues present early in life. The multicomponent LEIFc intervention involves maternal education on responsive feeding practices and mother-infant communication while using a family-guided caregiver coaching approach called SS-OO-PP-RR (“super,” Setting the Stage, Observation and Opportunities, Problem Solving and Planning, Reflection and Review) during feeding sessions [31,32]. The SS-OO-PP-RR approach was developed based on adult learning theory and has been successfully used with parents and childcare providers to promote development across domains in infants and young children [31-33]. For the LEIFc intervention, SS-OO-PP-RR was modified to be specific to communication during feeding to promote responsive feeding (Textbox 2). It is used as a guide for the interventionist during the coaching session but will also be used to evaluate the fidelity of the intervention. The objective measure of responsive feeding will occur through video recording of feeding sessions between mothers and infants. Videos will be coded before the intervention and then after 2 coaching sessions between the interventionist and the mother during a feeding session. The subjective measure of responsive feeding will be collected via a self-report questionnaire completed by mothers before and after the intervention study visits.

**The “super,” Setting the Stage, Observation and Opportunities, Problem Solving and Planning, Reflection and Review intervention.**

**Setting the Stage (SS)**

- Asks the caregiver to update how responsive feedings have been going since the last visit—listens, encourages caregiver reflection, and sets up problem-solving as needed
- Shares information related to responsive feeding—connects strategies to the child’s long-term health, well-being, and communication development
- Clarifies responsiveness strategies to use during the session (watching for hunger and fullness cues, using talk and touch during feeding, and stopping when the baby is full)

**Observation and Opportunities to Embed (OO)**

- Observes the caregiver-child interaction in feeding routine—provides feedback and builds on dyad strengths
- Uses coaching strategies (guided practice, direct teaching, demonstration, and caregiver practice) to help the caregiver embed responsiveness strategies into the feeding routine
- Provides general and specific feedback on caregiver and child behaviors and interactions (links caregiver strategy to child response)

**Problem-Solving and Planning (PP)**

- Problems solved with the caregiver about appropriate responsive feeding strategies
- Supports the caregiver to identify opportunities to help other caregivers use the strategies (other parents, childcare, and grandparents)

**Reflection and Review (RR)**

- Asks questions and comments to promote caregiver reflection and review of the feeding routine—identifies what worked for the caregiver and child
- Engages the caregiver to lead development of a “best plan of action” for using responsiveness strategies between visits

### Setting and Participants

Mothers of any parity will be recruited from community settings during the third trimester (28 weeks and beyond) of a healthy pregnancy. To be eligible, mothers need to be aged 18 years or older, able to read and speak English, and agree to video recording of an infant feeding in their home. Any medical or congenital condition in the fetus that would interfere with infant feeding or growth (ie, Down syndrome or cleft lip or palate) will be excluded. Mothers will be enrolled in the third trimester and then screened again after the birth of the infant to ensure that mother and infant remain eligible to participate.

An interventionist will be recruited to deliver the LEIFc intervention in the homes of new mothers. He or she will ideally have a background in maternal-child health and may be a nurse, social worker, speech-language pathologist, dietician, or early child development specialist. The interventionist will be trained on the SS-OO-PP-RR approach and the LEIFc protocol. Because the purpose of this study is to test the fidelity and feasibility of the intervention, feedback from the interventionist will be sought as part of the research protocol.

### Procedures

Eligible mothers will be identified through community settings such as obstetrician offices, prenatal parenting or birth classes, social media forums that pregnant moms may frequent, and word of mouth. If eligible and interested in the study, study personnel will consent mothers for the study. Once enrolled in the study, the interventionist will implement each study visit (Table 1). Study visit 1 is conducted when the mother is in the third trimester of pregnancy. Written material on infant feeding and infant cues of hunger and satiety is provided. Additionally, 2 short videos, one on infant hunger cues and the second on infant satiety cues, are watched with the interventionist and the mother. Any questions on feeding or communication are answered by the interventionist. Study visit 2 is conducted when the infant is approximately 1 month old. At this visit, a video recording of a feeding session between the mother and the infant is taken with no interaction with the interventionist. The feeding can be from the breast or from the bottle (with breastmilk or formula). Study visits 3 (at infant age of 2 months) and 4 (at infant age of 3 months) are the intervention study visits. At each visit, the interventionist will use the SS-OO-PP-RR tool to coach the mother through the feeding to identify hunger and satiety cues and discuss ways to respond. Each of these visits is also video recorded. The final study visit is at infant age of 4 months when another video recording of the infant feeding session is taken with no coaching from the interventionist.

At study visit 1, demographic data are collected along with the mother’s prenatal plans for infant feeding. At study visit 2, birth history is collected along with infant feeding practices, and the mother completes the Infant Feeding Questionnaire (IFQ). During study visits 3 and 4, infant feeding practices are collected. At study visit 5, the mother completes the IFQ and provides feedback on the study itself through a series of qualitative questions.

**Table 1:** Timing of study visits and procedures.

Study visit	Timing of visit	Components of the visit	Visit location
1	Prenatal (28 weeks or beyond of gestation)	Education provided on communication with your baby during feeding—videos and written material	Video conferencing platform (Zoom)
2	Infant age approximately 1 month	Complete Infant Feeding Questionnaire Video record the mother and baby during a feeding with no coaching Collect current infant feeding practices	Family home
3	Infant age approximately 2 months	Video record the mother and baby during a feeding Provide coaching to the mother during the feeding Collect current infant feeding practices	Family home
4	Infant age approximately 3 months	Video record the mother and baby during a feeding Provide coaching to the mother during the feeding Collect current infant feeding practices	Family home
5	Infant age approximately 4 months	Video record the mother and baby during a feeding with no coaching Complete Infant Feeding Questionnaire Collect current infant feeding practices	Family home

### Outcome Measures

The primary outcome in this study is the use of responsive feeding. This will be measured before (study visit 2) and after (study visit 5) intervention. Data are obtained subjectively through evaluation of the IFQ and objectively through a video review. The IFQ will provide a subjective measure of responsive feeding. This 20-item self-report tool has been validated for use in mothers of infants [29,34,35]. Likert-type scoring is used; higher scores indicate a stronger measure of the construct. Three subscales will be used to measure responsive feeding: use of food to calm, use of food to soothe, and awareness of infant cues.

The objective measure of responsive feeding will be collected via coding of the video session before and after intervention. The research team developed a coding scheme to capture both mother and infant behaviors during the feeding interactions. The coding scheme was adapted from the Responsiveness to Child Feeding Cues Scale [36] to define specific, discrete maternal behaviors that relate to responding to infant hunger and satiety cues as well as early infant communication acts. Infant behaviors are coded for hunger, satiety, and early communication acts. Research assistants will be trained on the coding scheme and will use existing videos to code with a goal of at least 85% agreement achieved to confirm inter-rater reliability. The coding scheme developed by the team will be used to code the feeding interactions for study visits 2 and 5. Videos will be coded for the number and rate of maternal utterances directed toward the child and responsiveness to the child’s vocalizations using the Noldus Observer XT software. The percentage of the interactions spent jointly engaged between the mother and the infant will also be coded.

Infant feeding practices, demographic characteristics (ie, maternal age, marital status, education level, and race and ethnicity), and maternal depression will be considered as covariates. Initiation and duration of breastfeeding and age at complementary food introduction will be extracted from data and tabulated into variables: duration of breastfeeding (any and exclusive) calculated in weeks and age at complementary food introduction calculated in weeks. Demographic data are collected at study visit 1, infant feeding practices at study visits 2 through 5, and maternal depression (via the Patient Health Questionnaire-9) at study visits 2 through 5.

### Statistical Analyses

Power analysis for the matched-pair *t* test was conducted. With the lower limit of the medium effect size of 0.5 [37], sample sizes of 27 and 36 provided 80% and 90% power, respectively, under 1-sided directional null hypothesis and the nominal significance rate of .05. We will aim to enroll 30 participants for this feasibility and fidelity study.

Once data are clean, descriptive statistics will be computed and examined. For the primary outcome, benefit will be determined by an increase in responsive feeding codes pre-post SS-OO-PP-RR coaching sessions (visits 2 and 5). IFQ subscale scores will be analyzed using matched sample *t* tests for pre-post coaching sessions to determine the benefit of the intervention. If the assumptions of statistical tests are not clearly met, the Mann-Whitney *U* test would be considered as an alternative. In a future pilot randomized controlled trial (RCT), analysis of covariance (ANCOVA) will be conducted where the difference between treatment and control groups for the postmeasure is examined with the premeasure as a covariate. The use of ANCOVA provides a statistical control in addition to the design control for the premeasure via random assignment in the RCT.

## Results

This study was initially designed to begin recruitment in the spring of 2020. Because of the COVID-19 pandemic, all research activities ceased from March to September 2020. The research team modified the protocol to conduct this study with socially distanced measures in place (ie, using videoconferencing platforms for data collection) and began recruitment in October 2020. Two enrolled participants were unable to complete study visits as scheduled, and data collection was unsuccessful. New funding was sought and obtained; recruitment began again in April of 2022 and data collection soon after. It is anticipated that data collection will be complete in April of 2023.

## Discussion

### Anticipated Findings

Although data collection for this study is ongoing, it is anticipated that the LEIFc intervention will be feasible to implement with and socially acceptable to mother-infant dyads by a trained interventionist. Additionally, it is hypothesized that fidelity of the intervention would need to be refined to better meet the needs of mother-infant dyads, especially those from high-risk populations (ie, minority groups and low socioeconomic status). Qualitative data collected from mothers (visit 5) and from the interventionist (visits 3 and 4) will be used to refine the intervention. Finally, it is hypothesized that there may be a change in responsive feeding before or after intervention; however, it cannot be attributed to the intervention until tested with a control group, in the future planned RCT.

### Implications

Childhood obesity rates continue to rise. Data from the National Health and Nutrition Examination Survey (NHANES) found that between 2017 and 2020 (prepandemic), 19.7% of children aged 2-19 years in the United States had obesity [38]. This is up from the previous NHANES data reporting 18.5% of children with obesity [39]. This rise in childhood obesity percentage suggests that efforts in recent years are not enough and more strategies are needed to halt this epidemic. Additionally, there are a higher percentage of children who are non-Hispanic Black, Hispanic, or from families living below the poverty line with obesity (24.8%, 26.2%, and 25.8%, respectively) [38]. Children with obesity are at risk for lifelong cardiometabolic conditions such as hypercholesterolemia, hypertension, and type 2 diabetes [40,41]. Children with obesity are also more likely to have mental health disorders (ie, depression and anxiety) and complications of the pulmonary, orthopedic, and gastrointestinal systems [1,42,43]. The first 1000 days of life, from conception to age 2 years, is a critical period for the development of habits that contribute to one’s weight status later in life, such as dietary preferences and behaviors [3,44-46]. Therefore, interventions implemented during the first 1000 days and targeting infants from high-risk groups (those of minority race and ethnicity and low socioeconomic status) have potential to decrease one’s obesity risk and subsequent complications. The promotion of responsive feeding is one such intervention that could be used to promote healthy infant weight gain and prevent obesity.

### Outcomes

Education on and promotion of responsive feeding should begin prenatally and evolve with the developmental of the child [5]. Research has demonstrated that responsive feeding during infancy helps an individual learn to eat in response to their own internal cues opposed to eating on a schedule [8-10]. The development of this eating pattern early in life may promote healthy eating behaviors lifelong, thus contributing to healthy weight status and prevention of cardiometabolic complications [41]. In addition, this early support for maternal-child communication may enhance language development during infancy. Infants’ earliest communication acts relate to signaling hunger cues, and maternal responsiveness to a child’s cues has long been established as an important predictor of later language outcomes [14]. As such, the LEIFc intervention has the potential to support 2 positive outcomes in children—healthy weight gain and positive communication outcomes.

Infant feeding in the first months after birth often occurs in the home, making this an ideal environment to promote responsive feeding [44,46]. Should the LEIFc intervention be successful, implementation in maternal-child home visiting programs would be sought. Maternal, infant, and early childhood home visiting programs, such as Early Head Start and Healthy Start, provide services to families from pregnancy up to the age of 5 years in at-risk communities [47,48]. These programs serve families from low socioeconomic backgrounds that often include those of minority status. Children from these families have higher rates of childhood obesity, cardiometabolic complications, and language delays [38,43]. The implementation of a responsive feeding intervention in these programs has potential to prevent several complications in children who receive this support, making implementation of the LEIFc intervention an important component to maternal-child home visiting program curricula.

### Conclusions

The current funded study will test the feasibility and fidelity of the LEIFc intervention in a group of mother-infant dyads. The intervention has potential to promote 2 childhood outcomes: healthy weight gain and early language development. The results of this feasibility study will allow the researchers to refine the intervention and move to phase IIa of the ORBIT model, testing proof of concept of the intervention. If successful, the LEIFc intervention would be expanded to include vulnerable mother-infant dyads enrolled in maternal-child home visiting programs. Once implemented into the curricula of such programs, application and sustainability would be strong.

## Appendix

Multimedia Appendix 1 External peer review comments from Florida State University College of Nursing.

## Abbreviations

ANCOVA: analysis of covariance
IFQ: Infant Feeding Questionnaire
LEIFc: Learning Early Infant Feeding Cues
NHANES: National Health and Nutrition Examination Survey
ORBIT: Obesity-Related Behavioral Intervention Trials
RCT: randomized controlled trial
SS-OO-PP-RR: “super,” Setting the Stage, Observation and Opportunities, Problem Solving and Planning, Reflection and Review

## Data Availability

Data are available from the corresponding author upon request.
