# Supplementary material for: Development of an Individualized Responsive Feeding Intervention—Learning Early Infant Feeding Cues: Protocol for a Nonrandomized Study
Source: JMIR Res Protoc. 2023 Feb 28;12:e44329. doi: 10.2196/44329 (PMC10015354; doi:10.2196/44329)
Supplement: Multimedia Appendix 1 [file resprot_v12i1e44329_app1.pdf]

From:
To:
Subject: RE: DRA Awards
Date: Monday, November 29, 2021 9:57:21 AM
Attachments: Proposal_r1.doc
_r2.doc
Critique_r3.doc

Attached are the individual comments from three reviewers. Congratulations again!!

The rating is intended to reflect the scientific merit of the proposed research against a scale of ideally constant characteristics.

Score	Descriptor	Additional Guidance on Strengths/Weaknesses
1	Exceptional	Exceptionally strong with essentially no weaknesses
2	Outstanding	Extremely strong with negligible weaknesses
3	Excellent	Very strong with only some minor weaknesses
4	Very Good	Strong but with numerous minor weaknesses
5	Good	Strong but with at least one moderate weakness
6	Satisfactory	Some strengths but also some moderate weaknesses
7	Fair	Some strengths but with at least one major weakness
8	Marginal	A few strengths and a few major weaknesses
9	Poor	Very few strengths and numerous major weaknesses

Best regards,

Associate Dean for Research & Professor

From:
Sent: Monday, November 29, 2021 9:54 AM
To:
Subject: DRA Awards

Dear Dr. ,

Congratulations! Your proposal "Testing Fidelity and Feasibility of a Responsive Feeding Intervention in a Group of Mother-Infant Dyads" has been selected for College of Nursing (CON) Dean's Research Award with the funding in the amount of $10,000. Please see the attached letter for more details. We truly appreciated your effort in informing us about your proposed research project. Please meet with the CON Grant Specialist () for post award support. They will help you plan expenditures, order supplies, and manage your budget.

PS: I will send a separate email including all the individual comments.

Best regards,

Title: Testing Fidelity and Feasibility of a Responsive Feeding Intervention in a Group of Mother-Infant Dyads

Principal Investigator(s):

OVERALL IMPACT

Reviewers will provide an overall impact score to reflect their assessment of the likelihood for the project to exert a sustained, powerful influence on the research field(s) involved, in consideration of the following five scored review criteria, and additional review criteria. An application does not need to be strong in all categories to be judged likely to have major scientific impact.

Overall impact: overall score = _3_

Strengths

- Preventing childhood obesity among Black families is an essential step in achieving health equity.
- Early interventions for children's eating/feeding, including helping parents understand infants eating and food-related cues, are essential.
- The PI is well-prepared to execute the described research, including a team of collaborators with complementary skills who have a history of collaboration.
- The PI describes an appropriate study design with generally well-described procedures and approaches.

Weaknesses

- The absence of an aim focused on intervention acceptability is a missed opportunity.
- Additional detail related to the videotaping and coding of the observational assessment would give greater confidence that high-quality data will be captured and handled appropriately.

SCORED REVIEW CRITERIA

Reviewers will consider each of the five review criteria below in the determination of scientific and technical merit and give a separate score for each.

1. Significance: score = _4_

Strengths

- **Major:** Culturally-tailored interventions in early life feeding/eating are needed to minimize racial inequalities in childhood obesity.
- **Moderate:** The proposed research is the next essential step. The investigator will ultimately need preliminary data to support extramural funding.

Weaknesses

- **Moderate:** In-home interventions of any type are exceedingly difficult to scale to achieve population impact ultimately.
- **Moderate:** It is not clear why the acceptability of the intervention is not studied in this pilot as it is critical to the research program.

2. Investigator(s): score = _1_

Strengths

- **Major:** PI has been systematically advancing this research, and she has surrounded herself by a competent team of collaborators with needed complementary expertise.
- **Moderate:** There is evidence the team has successfully worked together.

Weaknesses

- None noted

3. Innovation: score = _2_

Strengths

- **Moderate:** A relatively new intervention focused on helping parents identify and respond to infants' cues related to eating/feeding.
- **Minor-to-Moderate:** A combination of "objective" and "subjective" assessments.

Weaknesses

- None noted

4. Approach: score = _3_

Strengths

- **Moderate-to-Major:** The investigator describes an appropriate design, reasonably thorough description of procedures, clear description of critical measures.

Weaknesses

- **Minor-to-Moderate:** High-quality video and audio of the maternal feeding encounters at visits 2 and 5 are critical to project success. More detail on the positioning of the camera, filtering out background noise, and potential participant discomfort being videotaped during breastfeeding could give greater confidence that high-quality data will be obtained.
- **Minor-to-Moderate:** In lack fashion, more detail on the behavioral coding strategy, previous experience, and plans to maximize inter-coder agreement would give greater confidence these data will be appropriately handled.

5. Environment: score = _1_

Strengths

- A very detailed description of Environmental supports: the PI and team have access to sufficient human, scientific, and social supports to achieve the identified aims.

Weaknesses

- None

ADDITIONAL COMMENTS TO APPLICANT

Reviewers may provide guidance to the applicant or recommend against resubmission without fundamental revision.

Additional Comments to Applicant (Optional)
•

Title: Testing Fidelity and Feasibility of a Responsive Feeding Intervention in a Group of Mother-Infant Dyads

Principal Investigator(s):

OVERALL IMPACT

Reviewers will provide an overall impact score to reflect their assessment of the likelihood for the project to exert a sustained, powerful influence on the research field(s) involved, in consideration of the following five scored review criteria, and additional review criteria. An application does not need to be strong in all categories to be judged likely to have major scientific impact.

Overall impact: overall score = 2_

Strengths

- Major-An extremely important health challenge, disparities in pediatric obesity and life-long impact, that the PI has developed a credible intervention for.
- Major-The previous work by the PI and team has allowed development of a novel intervention, and the proof of concept research design is likely to be successful

Weaknesses

- Minor-Because of funding this remains a validation study and awaits further funding for an extensive efficacy study

SCORED REVIEW CRITERIA

Reviewers will consider each of the five review criteria below in the determination of scientific and technical merit and give a separate score for each.

1. Significance: score = _2_

Strengths

- Major-Identifies a significant problem with health disparities in pediatric obesity with 2-fold higher rates of obesity in non-Hispanic black and Hispanic children than with non-Hispanic white children; and associated cardiometabolic conditions that are life-long
- Identifies critical period for interventions that could modify weight status and reduce obesity during first 1000 days of life, and key approach to address "responsive feeding".
-

Weaknesses

-

2. Investigator(s): score = __ can't score

Strengths

- **No biosketch but team appears to be good, experience of PI in the area noted**

Weaknesses

-

3. Innovation: score = 2_
**Strengths**<br>• Utilizes and validates a home-based individualized responsive feeding coaching intervention, Learning Early Infant Feeding Cues (LEIFc), developed by the PI and her team. More likely a successful intervention than laboratory/clinic-based interventions<br>• Adds family guided coaching approach SS-OO-PP-RR (Setting the Stage, Observation and Opportunities, Problem Solving and Planning, Reflection and Review) for both the intervention method and data collection on success (validation)<br>**Weaknesses**<br>•

4. Approach: score = 3_
**Strengths**<br>• The PI and team have already guided a study through the design of the intervention and this proposal focuses on validation in (proof of concept) through guided feedback<br>• As this is a feasibility study the research design for a pre-post quasi experimental design with no control group is appropriate<br>• The utilization of videos to monitor feeding response with coding and analysis of feeding, along with the IFQ should yield appropriate data for evaluation<br>**Weaknesses**<br>• This is a proof of concept study and will require an extensive efficacy study with a well designed control group to assess strength of intervention on obesity index over a period of time

5. Environment: score = __
**Strengths**<br>•<br>**Weaknesses**<br>•

ADDITIONAL COMMENTS TO APPLICANT

Reviewers may provide guidance to the applicant or recommend against resubmission without fundamental revision.

Additional Comments to Applicant (Optional)
•

Title: Testing Fidelity and Feasibility of a Responsive Feeding Intervention in a Group of Mother-Infant Dyads

Principal Investigator(s):

OVERALL IMPACT

Reviewers will provide an overall impact score to reflect their assessment of the likelihood for the project to exert a sustained, powerful influence on the research field(s) involved, in consideration of the following five scored review criteria, and additional review criteria. An application does not need to be strong in all categories to be judged likely to have major scientific impact.

Overall impact: overall score = 2.5__

Strengths

- Addresses a significant social problem, childhood obesity prevalence, in diverse infant-mother dyads, focusing on disadvantaged groups.

Weaknesses

- Study design may overestimate impact of the intervention (fidelity, feasibility) because of absence of a control group.

SCORED REVIEW CRITERIA

Reviewers will consider each of the five review criteria below in the determination of scientific and technical merit and give a separate score for each.

1. Significance: score = _2_

Strengths

- Addresses a significant societal issue: childhood obesity with a well-piloted intervention program for mothers.

Weaknesses

- None observed

2. Investigator(s): score = _3_

Strengths

- The PI specializes in pediatric nursing. All investigators are experienced in working with the target populations.

Weaknesses

- Unsure of prior funding and publication experience based on the application. Having CVs for the investigators is needed to generate a rating.

3. Innovation: score = _2_

Strengths

- I don't have the background to assess how novel this program is and what other

programs like it have been adopted, but it seems well thought out.

Weaknesses

- None noted

4. Approach: score = _3_

Strengths

- Large sample size planned (50 dyads) that helps control for attrition, and well designed visit protocol.

Weaknesses

- Not having a control group weakens the value of the project in terms of applying for external funding. That is, the data are not going to be particularly compelling for reviewers. Taking video may change the responsiveness of the mothers to the intervention (being on their best behavior during a visit and filming) artificially stimulating adherence during a visit. A better design would use a control group and monitor height and weight gain in the control and experimental groups. That way lapses by mothers in unobserved periods could be monitored indirectly (e.g., if no differences are found between the control and experimental groups). The design was apparently tried already as funded by a CC grant but interrupted by Covid. In that part of the proposal, it was implied that the study should be conducted in "non-vulnerable mother-infant dyads to establish fidelity and feasibility." I didn't see screening criteria in the proposal to ensure that vulnerable dyads would be screened out for this intervention or discussion of "management of high risk aspects of the work" (doing it during Covid).

5. Environment: score = _2_

Strengths

- The group has had experience with recruiting the relevant population and FSU has a history of access through Head Start initiatives.

Weaknesses

- We are still in Covid restrictions for the FSU IRB and it would be good to know how interventionists would be recruited and trained to minimize transmission risk (in both directions). Why would this study succeed when the prior one funded by CC failed?

ADDITIONAL COMMENTS TO APPLICANT

Reviewers may provide guidance to the applicant or recommend against resubmission without fundamental revision.

Additional Comments to Applicant (Optional)

- The proposal looks about ready to modify for an efficacy trial and it wouldn't take too much effort to rewrite and send out for feedback. Has this been done already (perhaps that is the K23 to NICHD mentioned in the potential for extramural funding section)?
